# Preventing sedentary lifestyles among young children born with congenital heart defects: A feasibility study of physical activity rehabilitation after surgical or catheterization intervention

**DOI:** 10.1371/journal.pone.0284946

**Published:** 2023-08-18

**Authors:** Neya Ramanan, Suzie Lee, Gyaandeo Maharajh, Richard Webster, Patricia E. Longmuir

**Affiliations:** 1 Children’s Hospital of Eastern Ontario Research Institute, Ottawa, Canada; 2 Faculty of Science, University of Ottawa, Ottawa, Canada; 3 Division of Cardiology, Children’s Hospital of Eastern Ontario, Ottawa, Canada; 4 Faculty of Medicine, University of Ottawa, Ottawa, Canada; 5 Division of Cardiovascular Surgery, Children’s Hospital of Eastern Ontario, Ottawa, Canada; 6 Faculty of Health Sciences, University of Ottawa, Ottawa, Canada; PhD, PLOS, UNITED KINGDOM

## Abstract

**Background:**

Children with congenital heart disease (CHD) often have inactive lifestyles and motor skill deficits beginning in infancy. The least active infants continue to be the least active children at school age. Enhancing physical activity and motor development in infancy, at the time of CHD treatment, may prevent inactive lifestyle habits.

**Methods:**

All children being treated, through surgery or catheterization, for congenital heart disease are eligible if they are 3 to 72 months of age at enrollment. The Peabody Motor Development Scales (Version 2) and 7-day accelerometry (Actigraph GT9X Link) assess motor skills and physical activity prior to treatment and 7 weeks, 6 months and 12 months post-treatment. Participants are randomized 3:1 to intervention:control. Until 7 weeks post-treatment, intervention activities focus on regaining pre-treatment mobility and midline crossing. From 7 weeks to 6 months post-treatment, the intervention is individualized to each child’s assessment results and is parent-led, delivered at home and play-based.

**Conclusion:**

This feasibility study will provide essential data for a randomized controlled trial to evaluate play-based, parent-delivered interventions optimized to support age-appropriate physical activity and motor skills among young children with CHD. Preliminary intervention efficacy data will inform an evidence-based sample size calculation, optimize intervention timing, and identify hypotheses on the motor skill—physical activity connection and the impact of play-based, parent-led interventions during recovery from CHD treatment. Long-term, the goal is to optimize motor skill and active lifestyles among young children with CHD, enabling their healthy growth and development and enhancing childhood quality of life.

**Trial registration:**

**Clinical trials registration:** NCT04619745.

## Introduction

Active play is critically important for young children [1–5] as it is the foundation for childhood socialization, provides emotional, psychological and cognitive benefits [5] and is essential for childhood health [6], and biological and psychosocial development [1, 5]. Children with simple or complex congenital heart defects (CHD) are often less active [7], unable to achieve the 180 mins of daily activity recommended for the optimal health of young children [8], even when these children have age-appropriate motor skills [9, 10]. Highly inactive infants with CHD become the most inactive school age children [11], suggesting that an effective intervention to enhance active play should target children with CHD in infancy.

Although most older children and adults with simple or complex CHD are more sedentary than healthy peers [12–14], a small proportion of these patients lead active lifestyles [15, 16] and exercise [17], fitness [18, 19], or movement skill training [17] improves their performance. Therefore, their sedentary behavior is unlikely to result from physiological limitations of the cardiac diagnosis. The hypoactive lifestyles [14] among children with simple or complex CHD are suggested to result from limited self-efficacy for physical activity [20], parental overprotection [21] or perceptions of the child as fragile [14, 17]. Uncertainty naturally leads to caution, and eventually a more sedentary lifestyle. Given the difficulty in altering established habits, few interventions to enhance active lifestyles among older children with CHD have demonstrated long-term benefits [18]. Interventions that target young children are required so that active lifestyles can be established before sedentary lifestyle habits emerge.

The development of an intervention targeting physical activity in very young children with CHD would be highly novel, with several theoretical issues identified. Most importantly, it is necessary to determine whether parents would be willing to have their children participate. It is unclear whether parents of younger infants and those with less severe forms of CHD would be willing to enroll in a 6-month intervention at the time of treatment (i.e., recruitment feasibility). There is also evidence needed to support the feasibility, and parent and healthcare professional acceptance of completing recruitment and baseline assessments within the 4-to-6 week interval between scheduling of treatment and treatment date. Data on intervention efficacy and compliance, and the willingness of patients to be randomized to either intervention or control conditions are required to calculate an appropriate randomized controlled trial (RCT) sample size. Data on the time to contact, enroll, and assess patients, as well as the time required to develop and implement each child’s intervention are required in addition to data on withdrawal and ineligibility rates.

To prepare for an RCT examining the efficacy of a 6-month intervention during the treatment and post-treatment recovery phase of infants with CHD, this study is evaluating the feasibility of participant recruitment, data collection procedures and outcome measures, the acceptability and suitability of the intervention, and the resources required. We are also gathering preliminary efficacy evidence of response to the intervention, and professional and parent perceptions of study burden. This feasibility trial is also designed to reveal unforeseen problems and provide an opportunity to find appropriate solutions that would optimize the RCT design. We hypothesize that it will be feasible to recruit patients, collect data and perform the outcome measures of the intervention. We also hypothesize that parents will not feel burdened by the study. Furthermore, we hypothesize that the 6-month, home-based, parent-led, kinesiologist-designed physical activity program, completed immediately after surgical or catheterization treatment, may enable young children with CHD to achieve the recommended 180 minutes of daily physical activity.

## Methods

### Study design

**Fig 1:**
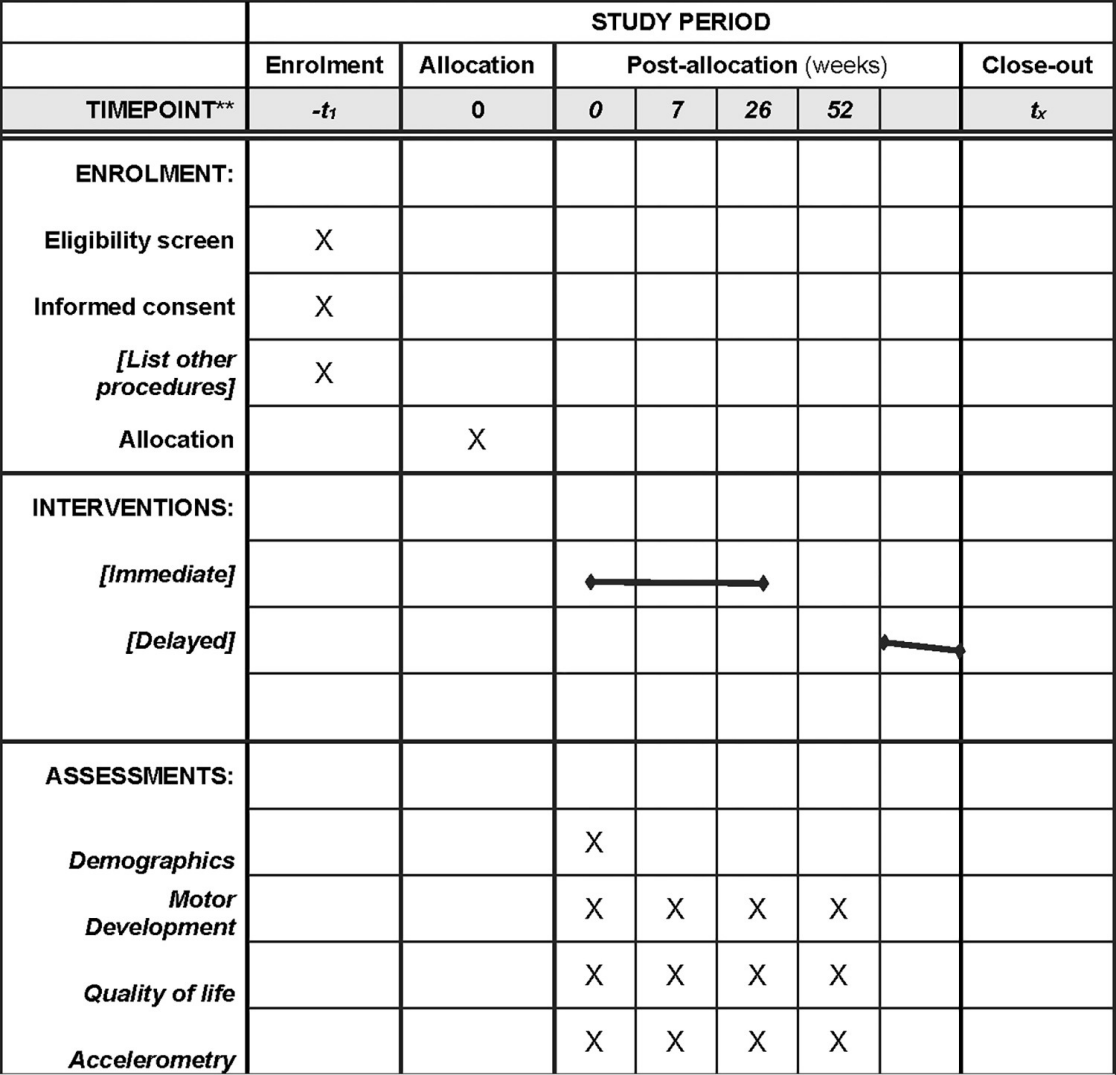
Schedule of enrollment, interventions, and assessments.

This hypothesis generating, feasibility study (Fig 1) is designed to include comprehensive measures of motor skill and physical activity, uniquely intervene at a very young age, and target the high-risk status for sedentary lifestyles of children with CHD. Long-term, the goal is to enable the design of a randomized, controlled trial to evaluate play-based, parent-delivered interventions optimized to support age-appropriate physical activity and motor skills among young children with CHD. Ethics approval for this study protocol (S1 File) is granted by the Research Ethics Board at the Children’s Hospital of Eastern Ontario (CHEO) (REB file #20/67X).

### Participants

All children receiving elective treatment via cardiac surgery or catheterization intervention for CHD at the Children’s Hospital of Eastern Ontario (CHEO) are eligible if they are 3 to 72 months of age. Patients with genetic conditions or physical disabilities affecting motor development, lack of independent limb movement, receiving medical care that is incompatible with the study assessments, or receiving emergency treatment are excluded. Total planned enrollment is 56 children, allowing for a sufficient sample (n = 48) to have 80% power to detect a difference in daily physical activity of 20% with alpha = 0.10 and allowing for a 15% drop-out rate.

Scheduled treatment procedures are reviewed weekly to identify eligible patients. Families are contacted by a member of the circle of care 4 to 6 weeks prior to the treatment (surgery or catheterization) date. Those willing to speak with the researcher are provided detailed information about the study. Families consenting to the child’s study participation provide written informed consent and complete the baseline assessment during the first study visit, with families choosing to have the visit in conjunction with the pre-treatment clinic visit or on a separate day. Given the young age of the participants, assent is assumed through the children’s willingness to perform the study activities. Some CHD diagnoses are more common in males, so recruitment of females is enhanced to target equal sex representation in the study sample. The cardiologist responsible for the child’s care approves their enrolment into the study and their continued participation prior to each study visit. The cardiologist also specifies any medically necessary activity restrictions that apply to the child. The approach and enrollment of patients is documented according to the CONSORT guidelines [22].

### Assessment of outcomes

All children receive standard of care for their treatment procedure and complete four study visits: 1 to 2 weeks prior to treatment, and 7 weeks, 6 months, and 12 months after treatment. After the first study visit, participants are randomized to either the intervention or control study group. Participants are randomly assigned, in random blocks of 4 and 8, in a 3:1 ratio to either the intervention (n = 36) or control (n = 12, standard care but no intervention) study group. Standard care includes the monitoring of heart function and treatment recovery but does not include physiotherapy or any support for motor development or physical activity.

The feasibility outcomes assessed address four aspects of study design:

1. Participant recruitment (# of patients treated per month, randomization to control or intervention, clarity of inclusion/exclusion criteria, ability of healthcare professionals to facilitate pre-treatment recruitment, reasons for refusal/ineligibility),
2. Data collection procedures (% of patients with completed pre-treatment data, # of days available prior to treatment for baseline data collection, % of parents able to complete child accelerometer wear for 7 days, % of control and intervention who complete all data sessions, frequency of missing data, study time and burden),
3. Intervention delivery (% retention and follow up, % compliant with intervention and rate of adherence, intervention time and burden), and
4. Required resources (staff time required to identify/consent/follow patients, space available for baseline testing, kinesiologist time to create/support interventions).

Preliminary efficacy data are collected from all study participants at or after each 1-hour study visit. The researcher conducting the study assessments is blind to study group allocation. The medical history of each participant is compiled from medical records (e.g., diagnosis, type and time of repair, length of hospital stay(s)). Parents complete four questionnaires: a) demographic questionnaire (age, gender, family income, parent education), b) an assessment of their child’s quality of life (Pediatric Quality of Life (PedsQL [23] or Infant Toddler Quality of Life (ITQOL-47)^54^), c) the Social Skills Checklist [24] for their child, and d) the Parenting Stress Index: short form [25]. 100mm visual analogue scales are used to evaluate parent perceptions of the child’s activity and skill relative to peers (above/below), activity and skill relative to optimal levels (above/below), and the perceived activity implications of the child’s CHD diagnosis (uncertain/clear) and treatment (very limited/none). Children 5 years of age or older also complete the PedsQL child report. All parents who do not complete the study and 10 randomly selected parents from those with complete data are asked to partake in a semi-structured exit interview, where they are asked about their perceptions, barriers, and facilitators to study completion. At each study visit, all child participants complete the Peabody Developmental Motor Scales-2 (test-retest ICC = 0.97 [26]) [27]. Children are asked to perform a series of play-based activities (building blocks, jumping, etc.) based on activities that could be completed by children of similar chronological age. The difficulty of the activities is then adjusted until the actual skills of the child being assessed are determined.

At the end of each study visit, children are given a tri-axial Actigraph GT9X Link accelerometer to wear on a waistband under the child’s outer garments, over the right hip, in the mid axillary line [28]. The daily physical activity of independently mobile children is measured over 7 consecutive days, with parents recording wear, nap, and sleep times, and reasons for device removal on a log sheet. At least 3 weekdays and 1 weekend day of valid data (at least 5 hours of recorded movement data excluding sleep and nap times [29]) is required [30]. The accelerometers measure activity intensity and established cut points [31] are used to calculate daily minutes of sedentary and light+moderate+vigorous activity.

### Intervention

Each child in the intervention group is provided with 6 months of parent-led, home and play-based activity plans that follow a standardized format and are individualized to each child’s age and previous visit assessments. The in-hospital intervention begins once the child returns to the regular hospital ward from the ICU, where the kinesiologist provides daily play activities that are focused on maintaining or regaining range of motion (upper and lower limbs) and supporting midline crossing (e.g., hand clapping, reaching for toys). The kinesiologist collaborates with parents to ensure accurate performance and implementation of the in-hospital program. From discharge to week 7, lower body mobility activities are combined with the in-hospital activities, since upper body weight bearing and lifting activities are restricted for children undergoing surgical treatment until the week 7 evaluation (for sternal healing). The kinesiologist contacts parents weekly to provide support, adjust the child’s physical activity, and assess adherence.

If physical activity is unrestricted after the week 7 assessment, progressively more difficult, individualized, parent-led, home and play-based activity plans (see S2 File) are provided every four weeks until the 6-month study visit. Each plan reflects the child’s baseline and week 7 assessment results and focuses on developing daily activity habits and age-appropriate movement skills. The kinesiologist demonstrates the planned activities and educates parents on implementing the activities through active play, as well as contacts parents every 2 weeks to provide support and obtain feedback on the child’s progress. At each contact, the kinesiologist assesses the ability of parents to properly lead the play-based activities and monitors home-based adherence to assess intervention fidelity.

### Data analyses

Feasibility outcomes (participant recruitment, data collection procedures, intervention delivery, RCT resources) are calculated as descriptive statistics and assessed relative to criteria established prior to study implementation. Based on our previous intervention trials with children with CHD, it is established that recruitment is considered feasible if at least 60% of families are willing to enroll and less than 20% of patients are ineligible, missed or withdrawn. Inclusion criteria are revised if more than 5% of patients required a cardiologist consult to determine eligibility. Data collection procedures are feasible if at least 80% of participants completed pre-treatment data, including accelerometer wear, and the scores from parents and healthcare professionals indicated that both the time and burden of data collection are acceptable (> 60 mm on visual analogue scale). Less than 20% of participants are to have missing data or data collection sessions during study participation. The intervention is feasible if less than 20% of families withdraw prior to study completion, and at least 70% of families complete the activities at least 3 times per week. The resources to conduct the study are feasible if adequate space is available for 100% of baseline testing sessions and the Research Coordinator has sufficient time to identify, consent, assess and intervene with all children receiving CHD treatment.

Frequency tabulations and descriptive statistics (mean±SD or median [q1, q3] as appropriate) are used to describe study participants. Logistic regression, adjusted for age, sex and group (intervention/control) is used for the preliminary assessment of study visit impact (1/2/3/4) on achievement of age-appropriate movement skills (Peabody Total Score < 1 SD from mean) or physical activity (180 mins/day). Groups are compared at each time point (chi-square). To examine group differences on changes in a) physical activity and b) motor skills, repeated measures ANOVA are used. Group (intervention/control) and time (baseline, 6 months, 12 months) are the within-repeated measures factor, with age, sex, and season as covariates. These analyses are not powered to determine statistical effects but will provide descriptive statistics for the feasibility study. Change in physical activity and motor skill effect sizes by study group indicating potential intervention impact, will be used to inform the required sample size for a fully powered multi-site randomized controlled trial and confirmed outcome variable changes are in the expected direction.

## Discussion

Pediatric CHD research has historically focused on reducing mortality. Now that even those with complex CHD survive to adulthood, the importance of promoting quality of life and decreasing secondary morbidity risk has dramatically increased. Through our unique and innovative research, we are striving to ensure that children with CHD are supported to not only survive, but thrive. Clinicians and families caring for children with CHD know they often are less skilled and less active than their peers. This study is designed to target the creation of active lifestyle habits among our youngest patients utilizing parent-led, home and play-based interventions modeled on similar interventions successfully used in older children [18, 19]. The feasibility and efficacy data generated through this project will enable the optimal design of a full-scale multi-site randomized controlled trial evaluating the impact of this physical activity intervention timed for the expected change in children’s physical activity as they are treated for their CHD. The novel aspects of this research include the comprehensive motor skill and physical activity measures, participants’ young age, and the high-risk status for inactive lifestyles of children with CHD. The lack of existing data on active play among infants during recovery from CHD treatment currently limits our ability to evaluate intervention effectiveness in an appropriately powered trial.

### Limitations

Known limitations for this research included the variability that occurs within standardized assessment protocols, the limited capacity of young children for completing assessment tasks, and the potential for recruitment bias among families choosing to enroll in a physical activity study. Our study team’s demonstrated skill in implementing the proposed assessments with very young children [3, 10] is expected to mitigate the impact of assessment and technical problems. To minimize recruitment bias, all eligible children treated at CHEO, either through surgery or catheterization, are approached to participate. Approaching all children decreases the risk of bias from active parents being more likely to volunteer for a physical activity study. The standardized template for creating activity plans and the database of activities linked to assessment results enable intervention delivery, with data on the required kinesiologist support informing the potential of the intervention as a standard of CHD treatment.

## Conclusions

This study is designed to provide essential feasibility data for a randomized controlled trial to evaluate play-based, parent-delivered interventions optimized to support age-appropriate physical activity and motor skills among young children with CHD. Preliminary intervention efficacy data will inform an evidence-based sample size calculation, optimize intervention timing, and identify hypotheses on the motor skill—physical activity connection and the impact of play-based, parent-led interventions during recovery from CHD treatment. Long-term, the goal is to optimize motor skill and active lifestyles among young children with CHD, enabling their healthy growth and development and enhancing childhood quality of life [32].

## Supporting information

S1 Checklist SPIRIT 2013 checklist: Recommended items to address in a clinical trial protocol and related documents*. (DOC)

S1 File Approved study protocol. (PDF)

S2 File Example of play-based physical activity intervention. (PDF)
