# Supplementary material for: Preventing sedentary lifestyles among young children born with congenital heart defects: A feasibility study of physical activity rehabilitation after surgical or catheterization intervention
Source: PLoS One. 2023 Aug 18;18(8):e0284946. doi: 10.1371/journal.pone.0284946 (PMC10437896; doi:10.1371/journal.pone.0284946)
Supplement: S1 File — (PDF) [file pone.0284946.s002.pdf]

PROTOCOL COVER PAGE

PROTOCOL NAME: Preventing Sedentary Lifestyles among Young Children Born with Congenital Heart Defects: A feasibility study of physical activity rehabilitation after surgical or catheterization intervention

PRINCIPAL INVESTIGATOR: Dr. Pat Longmuir, Senior Scientist

Children's Hospital of Eastern Ontario Research Institute

401 Smyth Road, Ottawa, Ontario, K1H 8L1

Phone: 613-737-7600 ext 3908

Email: plongmuir@cheo.on.ca

Co-Investigators: Dr. Gyaandeo Maharajh, Cardiovascular Surgeon

Children's Hospital of Eastern Ontario

401 Smyth Road, Ottawa, Ontario, K1H 8L1

Phone: 613-737-7600 ext : 3256

Email: GMaharajh@cheo.on.ca

Dr. Suzie Lee, Cardiologist

Children's Hospital of Eastern Ontario

401 Smyth Road, Ottawa, Ontario, K1H 8L1

Phone: 613-737-7600 ext: 3658

Email: SuLee@cheo.on.ca

Funded By: ***Heart and Stroke Foundation of Canada***

Current Protocol Version: ***November 16th, 2021***

1. Background Information and Scientific Rationale

1.1 Background Information

Physically Active Play is Critically Important for Children

Active play is critically important for young children[8, 11, 18-20] as it is the foundation for childhood socialization. It provides emotional, psychological and cognitive benefits[20] and is essential for childhood health[21], and biological and psychosocial development[8, 20]. Through active play, children develop skills, endurance, coordination, strength[22] and readiness for academic learning[23]. As such, physical activity is highly correlated with childhood health[20] and quality of life[9]. The physical health benefits of active lifestyles are widely recognized, including decreased atherosclerosis, diabetes and obesity risk[20, 21]. Active lifestyles also provide important emotional health benefits; improved perceived well-being, self-esteem, self-efficacy, and academic/cognitive performance[23, 24]. The evidence-based Canadian 24-Hour Movement Guidelines[14] recommend at least 180 minutes of physical activity per day in the early years (< 5 years of age)[14]. There is evidence that physical activity declines from 3 years of age[12], although Canadian children 18-59 months of age typically perform 232 (218, 246) minutes per day[25].

Children with CHD are More Sedentary

We measured daily physical activity among 127 children with CHD (58 female), 30±11 months of age, finding that their mean light + moderate + vigorous physical activity was 109±37 minutes per day. Children were enrolled from 1 to 3 years of age and followed to 5 years of age (HSFC grant #G-13-0002720, PI: Longmuir). We assessed children with CHD not requiring treatment (n=41), CHD treated without cardiopulmonary bypass (n=22), CHD treated with bypass (n=40) or an innocent heart murmur (n=24). At 12 to 18 months of age, the boys (n=11, 76-124 mins) and girls in our study (n=9, 92-149 mins) had activity levels that were similar to data for healthy infants (n=28, 94-142 mins[25]). However, the healthy comparison group was considerably younger (mean age 9.5 months (range 4 to 15)). Since activity levels increase with age in infancy (as children learn to walk and crawl), it is of concern that the older infants seen in our cardiac clinic had activity levels comparable to much younger children. Lower activity levels were also observed among our patients 18 to 59 months of age (boys: n=58, 141-163 mins; girls: n=49, 134-155 mins) compared to their healthy peers (n=232, 218-246 mins)[25]. Importantly, most of our patients did not achieve the 180 mins/day recommended for optimal health[14]. Some have suggested that parental overprotection is a contributing factor to these sedentary lifestyles[26].

Children with CHD are at Increased Risk for Sedentary Lifestyle Morbidities

Motor skill delays and inactive childhoods triple the risk of sedentary lifestyles[27]. Children with CHD have a high risk for motor skill delays[28, 29] affecting behaviour and visual-motor learning[30-34]. These deficits impact academic learning, communication and the perceptual- and visual-motor coordination needed for successful participation in active peer play. CHD treatment can also increase cardiovascular morbidity risk. For example, blood flow or coronary artery abnormalities increase atherosclerosis risk[35] and many CHD diagnoses are associated with systemic hypertension or decreased ventricular function[5]. Studies suggest that the abnormal myocardium among patients with CHD, and the ischemic stress during surgical procedures, may further increase their risk for hypertension, diabetes and obesity[5].

Children with CHD also have a high risk for psychosocial distress and reduced quality of life. Meta-analyses indicate psychological adjustment problems in 20% to 50% of patients; most commonly internalizing symptoms (anxiety, depression and withdrawal)[6, 7]. These concerns increase with age, and we have demonstrated that they are associated with reduced emotional quality of life[36]. Unfortunately, the hypoactive lifestyles of children with CHD limit their access to the mental health benefits provided by physical activity[37]. Given sedentary lifestyles[38], motor skill deficits[39] and psychological problems[6, 7] in children with CHD and considering the emerging evidence

of a physical activity->heart health->quality of life relationship, **enabling physically active lives and age-appropriate motor skills among young children with CHD is extremely important**.

Children with CHD Could be Active

Although most older children and adults with simple or complex CHD are more sedentary than healthy peers[28, 40, 41], exercise[42], fitness[16, 43], or movement skill training[43] improves their performance. Objective measures consistently indicate a small proportion (5% to 10%) of children with CHD, even those with the most complex form of CHD[44], achieve the daily physical activity recommended for their age[4]. Given they respond to interventions and those with complex CHD can achieve the recommended daily physical activity, we expect their sedentary lifestyles are not solely attributable to their heart malformation. **We hypothesize CHD treatment and limitations reduce active play opportunities, delaying motor skill development[8, 11, 18-20] and preventing active lifestyle habits** from being established in early childhood.

Changing Parent and Child Perceptions

The hypoactive lifestyles[41] among children with simple or complex CHD are suggested to result from limited self-efficacy for physical activity[45], parental overprotection[26] or perceptions of the child as fragile[41, 42]. Increasing activity self–efficacy through rehabilitation is recommended for these children[46]. Our research[47] suggests **children and parents are often uncertain about appropriate physical activity** for the child with CHD. Uncertainty naturally leads to caution, and eventually a more sedentary lifestyle. We hypothesize that the time of CHD treatment is particularly sensitive for an intervention targeting physical activity habits because activity uncertainty peaks as activity limits are expected to change, highlighting the need for enhanced physical activity support. Parents expect the child's capacity for physical activity will change after treatment, but remain uncertain of how/when it will change or how treatment/recovery will impact the child. Since uncertainty and overprotection are not limited to children with the most complex forms of CHD, we also hypothesize that it is important to examine the physical activity and motor skill of all children with CHD, irrespective of the specific heart defect.

Sedentary Lifestyle Habits are Difficult to Change

Although childhood physical activity begins to decline at 3 to 5 years of age[12, 13], interventions to enhance active lifestyles among older children have had little to no effect[48]. Duppen and colleagues[49] found no change in daily physical activity despite improvements in cardiorespiratory fitness in their sample of adolescents and young adults with CHD. A 1-year physical activity intervention implemented by Longmuir et al[43] initially increased daily physical activity. However, 12 months post-intervention activity had declined, although not to the extent expected with participants' increasing age. With evidence that activity tracks from childhood to adolescence[11], interventions are required that target very young children so that active lifestyles can be established before sedentary lifestyle habits emerge.

1.2 Rationale for this Study

Physically Active Lifestyles are Critically Important for Children with CHD

Twelve per 1000 children, or 3,500 Canadian children born each year[1], live with congenital heart defects (CHD)[2]. Treatment strategies are now exceptionally successful with over 90% of infants with CHD surviving to adulthood[3]. **Given this dramatic reduction in mortality, our focus must switch from enabling survival to helping these children thrive!** Thus, research addressing important morbidities must be given the highest priority. Older children and adults with simple or complex CHD typically have hypoactive lifestyles[4] and higher rates of diseases associated with sedentary lifestyles[5], such as atherosclerosis, anxiety and depression[5-7]. Since childhood physical activity is essential for normal development[8], provides the foundation for childhood

socialization and quality of life[9], and habits track from childhood throughout life[10], ensuring active lifestyles is critically important for children with CHD.

Sedentary Lifestyle Habits of Children with CHD Start in Infancy

Childhood physical activity decreases with increasing age[11], with the decline beginning as early as 3 years of age[12, 13]. Our 5-year observational study of physical activity and motor skill in young children with CHD (HSFC grant #G-13-0002720, PI: Longmuir) suggests **they do not achieve the 180 mins of daily activity recommended for optimal health**[14] even with age-appropriate motor skills (manuscript in preparation). Longitudinal analyses indicate that physical activity at the first study visit was strongly associated with activity at subsequent study visits. That the most sedentary children assessed in infancy continue to be the most sedentary children at school age suggests that **an effective intervention to enhance active play and prevent sedentary lifestyles must target children with CHD in infancy**.

Optimal Timing of an Intervention

We hypothesize that intervening during the treatment (via surgery or catheterization) and post-treatment recovery phase would be optimal for changing physical activity habits among young children with CHD. Prior to treatment, activity limits are clear. The dramatic extent of change after treatment and the need for added limits early in recovery can lead to parent uncertainty about the child's capacity for physical activity. Providing an intervention as part of treatment recovery would support parents through these changes, enabling them to establish appropriate active lifestyle expectations and habits for their children and emphasizing that activity is not only safe and appropriate, but also very important for their child's recovery and health.

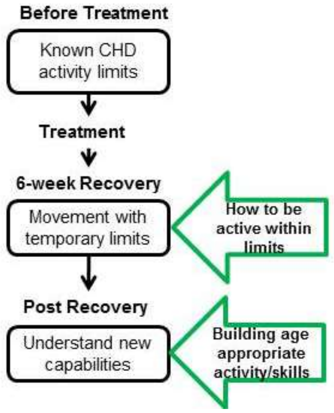

We have shown a post-operative physical activity program, implemented through parent-led exercise done at home, enables children with CHD to achieve normal fitness levels, regardless of defect severity or type of surgery (repair or palliation)[15]. These normal fitness levels were maintained 5 years post- intervention[16]. Importantly, the home-based program benefits accrued even if the child performed the exercises only 1 or 2 days per week; suggesting that the long-term fitness benefits were likely due to changes in habitual activity or perceptions of the child's capacity for exercise rather than physiological exercise training effects. However, these studies were completed when CHD treatments were performed after 5 years of age. Currently, treatment occurs very early in life, typically prior to 1 year of age for surgeries or prior to 3 years of age for treatments via cardiac catheterization. Although we have shown that parents of children with CHD 12 to 24 months of age are able to implement play-based interventions at home[17], that pilot study focused on age-appropriate motor development so the effectiveness of such an intervention for promoting recommended daily physical activity is unknown.

Gaps in Knowledge Prevent RCT Planning

Although we hypothesize that a 6-month intervention at the time of CHD treatment would effectively enhance the physical activity habits of these young patients, we currently lack the data required to plan a randomized controlled trial of such an intervention. Parents enthusiastically volunteered for our pilot study of a parent-led intervention on the motor development of young children with CHD[17], with the trial being fully enrolled less than 1 week after trial information was disseminated by cardiologists. However, that study targeted only children 12 to 24 months of age with very severe forms of CHD who are known to experience substantial motor skill deficits. In addition, the intervention was limited to 10 weeks, the study required only baseline and end-of-intervention assessments and families were not involved in CHD treatments at the time of

study participation. We do not know if parents of younger infants and those with less severe forms of CHD would be willing to enroll in a 6-month intervention at the time of treatment (i.e., recruitment feasibility). We also require evidence to support the feasibility, and parent and healthcare professional acceptance of completing recruitment and baseline assessments within the 4-6 week interval between scheduling of treatment and treatment date. Data on intervention efficacy and compliance, and the willingness of patients to be randomized to either intervention or control conditions are required to calculate an appropriate RCT sample size. Data on the time to contact, enroll, and assess patients, as well as the time required to develop and implement each child's intervention are also required.

2. Study Objectives

Feasibility Outcomes to Generate RCT Hypotheses

This feasibility study will provide the data required to conduct a randomized controlled trial of a post-operative physical activity intervention among infants and young children with CHD. The research question for the RCT would be: Does a 6-month, home-based, parent-led, kinesiologist-designed physical activity program, completed immediately after surgical or catheterization treatment, enable young children with CHD to achieve the recommended 180 minutes of daily physical activity?

To prepare for the RCT examining intervention efficacy, this study will evaluate the feasibility of participant recruitment, data collection procedures and outcome measures, the acceptability and suitability of the intervention, and the resources required. We will also gather preliminary efficacy evidence of response to the intervention, and professional and parent perceptions of study burden. This feasibility trial is also designed to reveal unforeseen problems and provide an opportunity to find appropriate solutions that would optimize the RCT design.

2.1 Primary Feasibility Objectives

To assess the feasibility of conducting a RCT to determine the impact of a 6-month physical activity intervention immediately after CHD treatment, we will determine the following metrics:

1) <u>Feasibility of participant recruitment</u>:
a) # of patients treated per month, % ineligible, % willing to enroll, % missed, % who withdraw,
b) willingness of parents to be randomized to either intervention or control study groups,
c) inclusion/exclusion criteria clear and sufficient or too inclusive or restrictive,
d) healthcare professionals ability to facilitate pre-treatment recruitment,
e) reasons for study refusal or ineligibility.

2) <u>Data collection procedure feasibility</u>:
a) % of patients with complete pre-treatment data,
b) time available prior to treatment for baseline data collection,
c) % of parents able to complete child accelerometer wear for 7 days,
d) % of control and intervention patients who complete all data collection sessions,
e) frequency of missing data due to medical condition/treatment, child cooperation, or other reasons,
f) parent and healthcare professional perceptions of time and burden for data collection.

3) <u>Feasibility of physical activity intervention</u>
a) retention and follow up rates for the intervention group,
b) % compliant with intervention and rate of adherence (# of sessions per week),
c) parent perceptions of the appropriateness, time and burden of the intervention.

4) Resources to conduct the RCT
a) staff time required to identify, consent and follow patients and perceived burden of study activities,
b) space available for baseline testing,
c) kinesiologist time required to create and support parents in leading individualized interventions.

2.2 Secondary Efficacy Objectives

We will conduct a preliminary assessment of efficacy outcomes using the following metrics:

5) Promise of intervention
a) Time required after cardiac treatment intervention for child to achieve age-appropriate motor skills (Peabody Developmental Motor Scales – 2),
b) Examination of participant-level activity data suggest the intervention is likely to be successful,
c) Changes in outcome variables are in the expected direction,
d) Estimates of effect size suggest intervention has promise.

6) Sample size calculations
a) Change in daily minutes of physical activity from before the cardiac treatment intervention to the achievement of age-appropriate motor skills (7-day accelerometry)
b) Change in motor skill and daily physical activity for 6 months after achievement of age-appropriate motor skills (i.e., end of intervention)

3. Eligibility Criteria

3.1 Inclusion Criteria

a) Female or male at least 3 months of age but not more than 72 months of age (upper age limit for valid Peabody Motor Development Scales-2 assessment)
b) Receiving elective treatment via cardiac surgery (Dr. G. Maharajh) or catheterization intervention (Dr. S. Lee) for CHD at the Children's Hospital of Eastern Ontario.

3.2 Exclusion Criteria

a) Genetic conditions or physical disabilities impacting motor development (e.g., Down syndrome)
b) Emergency treatment for child in critical condition
c) Medical care not compatible with study assessments
d) No independent limb movement.

4. Study Design

This feasibility study includes comprehensive measures of motor skill and physical activity, uniquely intervenes at a very young age, and targets the high risk status for sedentary lifestyles of children with CHD. The lack of existing data or previous research on active play among infants during recovery from CHD treatment limits our ability to evaluate intervention effectiveness in an appropriately powered RCT. This study will provide essential data on patient recruitment, data collection procedures, the proposed physical activity intervention and resources required to enable the design of an RCT to evaluate play-based, parent-delivered interventions optimized to support age-appropriate physical activity and motor skills among young children with CHD.

5. Expected Duration of Subject Participation

Each participant will be enrolled for 12 months.

6. Study Procedures and Evaluations

Based on hospital records, we expect 85 children will have cardiac surgery and 15 children will have a catheterization intervention during the study recruitment period. Of these, 80% will be less than 72 months of age. In our previous studies > 70% of families agreed to enroll, thus providing an initial sample of 56 study participants. In our intervention studies < 15% have withdrawn, therefore we expect complete feasibility data for 48 participants to conduct our assessment. Participants will be randomly assigned, in random blocks of 4 and 8, in a 3:1 ratio to the intervention (n=36) or control (n=12, standard care but no intervention) study group and documented according to the CONSORT guideline[50].

6.1 Participant Identification & Recruitment

The research team will review the list of scheduled treatment procedures weekly, as the lists become available, to identify patients who meet the study eligibility criteria. Eligible participants will be contacted by a member of the circle of care when the treatment date (surgery or catheterization) is scheduled; 4 to 6 weeks prior to the procedure. The clinical staff will ask parents if they are willing to speak with the researcher after details of the treatment date/plan are provided. Alternatively, the study information can be sent by mail (Appendix Q), and then the research team will follow-up with a phone call to eligible families, two weeks after the letter is sent. If parents agree, the researcher will contact them to provide information about the study (Appendix H) and will send them a copy of the consent (Appendix A) if desired. If parents' consent to the child's study participation, written informed consent for the research will be obtained during the first study visit, which will occur in conjunction with the pre-treatment clinic visit (i.e., families do not have to make an extra trip to CHEO for the research study assessment). Given the young age of the participants, willingness to do the study activities will indicate assent.

All children enrolled in the study will receive the standard of care for their treatment procedure and complete four study visits. Study visit #1 (consent, baseline assessment) will occur during the mandatory pre-treatment clinic visit, which is typically 1-2 weeks prior to treatment. Visits #2, #3, and #4 will occur 7 weeks and 6 and 12 months after treatment, respectively. After visit #1 is complete, children will be randomized to either the intervention or control study group. Control participants will follow the same schedule of assessments but no intervention will be provided.

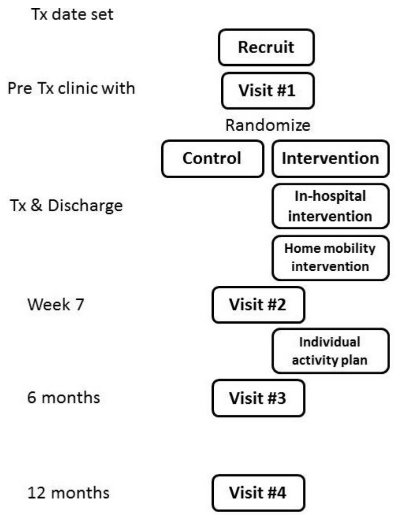

The week 7 post-intervention visit corresponds to a regular post-treatment follow up appointment and is also when post-operative activity restrictions (which allow the sternum to heal) are removed. The 6-month visit, also often corresponding to a follow-up in clinic, will evaluate motor skill and physical activity changes at the end of the individualized physical activity intervention. The final assessment, 12 months after treatment, will provide preliminary efficacy data for the sustained impact of the intervention.

The intervention group will complete individualized, parent-led, home and play-based activity plans for 6 months, beginning as soon as the child returns to the inpatient unit (i.e., not in ICU). While in hospital, the kinesiologist will deliver the intervention daily, encouraging child/parents to enhance limb range of motion, midline crossing, and recovery of pre-treatment mobility. Prior to discharge, the kinesiologist will give the parents a home mobility

intervention to follow until the week 7 visit. The home mobility intervention will be similar to the in-hospital activities with the addition of motor skill development activities that do not require upper body weight bearing or lifting and are appropriate to the child's baseline assessment results. The kinesiologist will contact parents weekly to provide support and assess adherence. At the week 7 visit, the first four weeks of the individualized activity plan will be provided. It will be designed to enhance the child's movement skills and daily activity beyond their week 7 assessment results. The kinesiologist will demonstrate the planned activities and educate parents on implementing the activities through active play. After the week 7 visit, the kinesiologist will contact parents every 2 weeks to provide support and obtain feedback on the child's progress. New individual activity plans will be provided every four weeks until the 6-month study visit. The kinesiologist will assess the ability of parents to lead the play-based activities as intended at each visit and monitor home-based adherence to assess intervention fidelity.

All children (intervention and control) will complete all outcome measures at or after each 1-hour assessment visit. Children will be given an omni-directional accelerometer to wear on a waist-worn belt for 7 days after each visit, including 2 weekend days, to assess daily physical activity. Parents will be requested to position the accelerometer over the right hip, in the mid axillary line. Movement skills will be assessed with the Peabody Motor Development Scales (version 2). Parents will complete a demographic questionnaire, the Pediatric Quality of Life Inventory (PedsQL[51]) proxy report for toddlers/young children (Appendix I, K, L), the Social Skills Checklist[52](Appendix J), Parenting Stress Index: short form[53] (Appendix E), and parents of children 4 years or younger will complete the Infant Quality of Life assessment (Appendix M). 100mm visual analogue scales will be used to assess parent perceptions of the child's activity and skill relative to peers (below/above), activity and skill relative to optimal levels (below/above), and activity implications of the child's CHD diagnosis and treatment (uncertain/clear, very limited/none). The child's medical history will be extracted from the medical record (Appendix F).

6.2 Assessments

<u>Motor Development</u>: Children will be evaluated with the Peabody Developmental Motor Scales – 2[54], a valid and reliable (test-retest ICC = 0.97[55]) measure of motor development from birth to 5 years of age that is sensitive for detecting change over time[55]. The child is asked to perform a series of play-based activities (building blocks, reading books, jumping, etc.) beginning with activities that a child of similar chronological age would be expected to perform successfully. The activities are then made progressively more or less difficult until the actual skills of the child are determined. The Peabody has previously been used to assess children with heart problems[17, 55].

<u>Demographics and Parent Perceptions</u>: Cardiac treatment history will be compiled from medical record reviews. Data extracted will include diagnosis, age at and time since repair, type of repair, length of hospital stay(s), frequency and type of complications/hospitalizations, current medications, and any physical activity restrictions. Parents will complete five questionnaires:
1) demographic (age, gender, # of siblings, family income, parent education, child illness/hospitalization or medication/treatment changes outside of our institution, language spoken at home)
2) Pediatric Quality of Life Inventory (PedsQL) Parent Proxy Report for Toddlers (standardized measure of physical, emotional, social and school/daycare quality of life widely used with children with CHD[51]). Children 5 years of age and older will also complete the Peds QL child report (Appendix K).
3) Social Skills Checklist[52] to assess the child's skills and interactions with peers
4) Parenting Stress Index: short form[53] (PSI) as a measure of parent stress during the child's treatment and recovery.
5) Parents of children 4 years and younger will complete the Infant Quality of Life questionnaire (ITQOL-47; Appendix M)

Children 0-4 years of age questionnaires: Demographic, Peds QL Parent Report, Social Skill Checklist, Parenting Stress Index and Infant Quality of Life.

Children 5-6 years of age questionnaires: Demographic, Peds QL Parent Report, Peds QL Child Report, Social Skill Checklist and Parenting Stress Index.

We will use the demographic questionnaire developed for our longitudinal study (HSFC grant #G-13-0002720, PI: Longmuir) to obtain parent perceptions regarding the child's physical activity participation (Appendix B). Parent reported activity restrictions will be compared to medical record activity restrictions as an index of overprotection. Scoring of the PedsQL, Social Skills Checklist, Parenting Stress Index and ITQOL-47 will follow published procedures. All parents who do not complete the study and 10 parents randomly selected from those with complete data will be asked to complete an exit interview. The semi-structured interview guide will ask for the positive aspects of the study and how it could be improved. The interviews will be audio-recorded and transcribed for inductive qualitative analysis to better understand their perceptions, barriers and facilitators to study completion.

Physical Activity: Each child will wear an Actical Z series accelerometer (memory > 190 days) on a waistband under the child's outer garments[56]. The goal is accelerometer wear 24 hours per day for 7 consecutive days to minimize potential data loss (monitor forgotten, activity before or after a nap, etc.). The Actical Z series can be worn for swimming or bathing. Parents will record wear, nap and sleep times, and reasons for device removal on a log sheet (Appendix G). Established cut points[57] will be used to calculate daily minutes of sedentary and light+moderate+vigorous activity. At least 3 weekdays and 1 weekend day of valid data (at least 5 hours of recorded movement data not including sleep and nap times[58]) will be required[59]. Accelerometers measure activity intensity in children without[25] and with heart problems[60], distinct from emotional or medication influences on heart rate. Children not yet independently mobile (i.e., cannot roll over, crawl or walk) will not wear an accelerometer but will complete the Peabody Motor Development Scales-2

6.3 Intervention

Each child receiving CHD treatment randomized to the intervention group will be provided with 6 months of parent-led, home and play-based activity plans. The activities in the plan will be tailored to each phase of treatment (in hospital, discharge to week 7, week 8 to 6 months), follow a standardized format and provide content individualized to each child's age and previous visit assessments.

In-hospital: The in-hospital intervention (see Appendix C for sample plan) will begin when the child returns to the regular hospital ward from the ICU. Play activities will focus on maintaining or regaining range of motion (upper and lower limbs) and supporting midline crossing (e.g., hand clapping, reaching for toys). Resumption of the motor skills and mobility demonstrated prior to treatment will be encouraged once all treatment equipment (e.g., chest drains) has been removed.

Discharge to Week 7: A range of motion and mobility intervention will be provided. It will be similar to the in-hospital plan but with the addition of lower body mobility activities to encourage movement skill development. Upper body weight bearing and lifting activities are restricted for children undergoing surgical treatment until the week 7 evaluation (for sternal healing). The kinesiologist will monitor/adjust the child's physical activity on a weekly basis until activity is unrestricted (i.e., after week 7).

Week 7 to 6 Months: Once physical activity is unrestricted, parent-led, home and play-based weekly plans (effective in our previous research[17]) will be designed by the kinesiologist to encourage active lifestyle habits (see Appendix D). Each plan will be individualized to the child's age and assessment results. A new set of progressive activity plans will be provided every four weeks until the end of the intervention (6 month visit), using parent feedback regarding the child's progress and the attainment of age-appropriate motor skills. The

kinesiologist will also educate parents about their child's assessed and desired level of daily physical activity and support parents as they implement the activity plans at home.

Kinesiology Support: Ms. Miranda DiGasparro, Kinesiologist and Research Assistant, will see children daily during their hospitalization, working with parents to implement the child's personal activity plan. Collaborative implementation will enable the kinesiologist to ensure accurate performance of target movements and answer parent questions. From discharge to week 7, the kinesiologist will contact parents weekly to answer questions and adjust the activities as needed to ensure they remain appropriately challenging and interesting as the child recovers, grows and develops. After the week 7 visit, Ms. DiGasparro will prepare individualized activity plans that progress activity type and difficulty. Each plan will reflect that child's baseline and week 7 assessment results and the goals of developing recommended daily activity habits and age-appropriate movement skills. The kinesiologist will contact the family every two weeks to obtain feedback on each individualized plan and make adjustments to the plans as necessary. A new set of four weekly plans will be sent to the family each month until the 6-month study assessment.

6.4 Feasibility Study Timelines

**Study Activity**	**Jul 2020 to Jun 2021**				**Jul 2021 to Jun 2022**				**Jul 2022 to Jun 2023**			
Ethics Approval	■											
Recruitment		■	■	■	■	■						
Intervention		■	■	■	■	■	■	■				
Assess #1 (base)		■	■	■	■	■						
Assess #2 (wk 7)		■	■	■	■	■	■					
Assess #3 (6 mo)				■	■	■	■	■				
Assess #4 (post)						■	■	■	■	■		
Data Analyses								■	■	■	■	
RCT Design									■	■	■	■

7 Potential Risks & Benefits

7.1 Risks

There are no known ethical barriers or potential harms recognized for this study as the assessment activities replicate childhood play. The Peabody Developmental Motor Scale – 2 (Peabody), is a valid and reliable measure of motor development from birth to 5 years of age[22], that is comprised of a series of play-based activities (building blocks, reading books, jumping, etc.) that the child is asked to perform. All assessments have previously been used, by ourselves and others, among children with simple and complex CHD. Accelerometers have also been extensively used to measure the physical activity of young children, including toddlers, and are approved by Health Canada as safe and effective for hospital use.

Patient participation will not require any change to the child's medical treatment. The cardiologist responsible for the child's care will approve their enrolment into the study and their continued participation prior to each study visit. The responsible cardiologist will also specify any medically necessary activity restrictions that apply to the child.

The potential discomforts associated with this study are primarily related to the demands of the motor skill assessments. During each assessment, children will be asked to perform tasks expected for their age, as well as tasks expected for younger and older children, in order to properly evaluate their current level of development. We anticipate that some parents may be uncomfortable watching their child attempt tasks that the child is not

yet maturationally-ready to perform. Children may also experience some discomfort if they are unable to perform an activity successfully (e.g., unable to retrieve a toy of interest).

We do not anticipate potential harms or discomfort associated with the wearing of the accelerometer. Actical accelerometers are waterproof to a depth of 10 metres, and therefore are safe for bathing, swimming and water play. The Actical is designed for use during high intensity physical activity. The force required to crack the sealed case would be well beyond what a child could generate. The belt for wearing the accelerometer will be individually sized for each child at each study visit to ensure that there are no loose ends or sections of strap that could pose a choking or strangulation hazard. The accelerometer belt can be worn over the child's under clothes, minimizing the potential for skin irritation from the strap rubbing during movement.

The inconveniences associated with participating in this research include the time required to attend study visits, the effort required to have the child wear the accelerometer belt and then return the accelerometer by mail after each assessment, and the time required to answer the parent questionnaires. Those randomized to the intervention group will also be required to complete the play activities with their child.

We anticipate that there will be no potential harms associated with participation in this project. There are no known consequences for children or families who choose not to participate in this research.

The discomforts associated with being approached to participate in the study may include the perception that non-participation would negatively impact current or future care. In order to address this issue, each family will be clearly told that their decision regarding whether to participate is entirely voluntary, that choosing not to participate will have no impact on their current/future care by their physician, other collaborating cardiologists or anyone at CHEO.

7.2 Benefits

The participants may benefit from the satisfaction of knowing that they are contributing to our understanding of physical activity among children with CHD. It is also possible that study participants may benefit in the longer-term, if the intervention is successful and aids in their achievement of a healthy, active lifestyle. Understanding their limitations and abilities will better support children with CHD and their families to adopt healthy, active lifestyles.

Participants will be reimbursed for the parking costs associated with the study assessment visits. Upon completion of the study participants will be given a $50 gift card, an End of Study Letter (Appendix R) in appreciation of their time and effort, their child's results of their study participation (Appendix S), and an explanation of the assessment results (Appendix T).

8 Statistical Plan

8.1 Sample Size Determination

Based on hospital records, we expect 85 children will have cardiac surgery and 15 children will have a catheterization intervention during the study recruitment period. Of these, 80% will be less than 72 months of age. In our previous studies > 70% of families agreed to enroll, thus providing an initial sample of 56 study participants. In our intervention studies < 15% have withdrawn, therefore we expect complete feasibility data for 48 participants to conduct our assessment. Participants will be randomly assigned, in random blocks of 4 and 8, in a 3:1 ratio to the intervention (n=36) or control (n=12, standard care but no intervention) study group and documented according to the CONSORT guideline[50].

Change in physical activity and motor skill effect sizes by study group will indicate potential intervention impact, be used to calculate sample size for a fully-powered RCT, and confirm that changes in outcome variables are in the expected direction.

8.2 Feasibility Outcome Assessment and Analyses

Data from the total population treated for CHD at our site (medium patient load) over the study recruitment period will be used to inform the design of the planned future multi-centre RCT. The Research Coordinator will track all study details and patient interactions, including participation changes, information inquiries or time required. Feasibility outcomes (participant recruitment, data collection procedures, intervention delivery, RCT resources) will be assessed and calculated. Based on our previous intervention trials with children with CHD, recruitment will be considered feasible if > 60% of families are willing to enroll and < 20% of patients are ineligible, missed or withdrawn. Inclusion criteria will be revised if > 5% of patients require an MD consult to determine eligibility. Data collection procedures will be feasible if > 80% of participants have complete pre-treatment data, including accelerometer wear, and the scores from parents and healthcare professionals indicate that both the time and burden of data collection are acceptable (> 60 mm on visual analogue scale). Less than 20% of participants will have missing data or data collection sessions during study participation. The intervention will be feasible if < 20% of families withdraw prior to study completion, and > 70% of families complete the activities at least 3 times per week. The resources to conduct the RCT will be feasible if adequate space is available for 100% of baseline testing sessions and the Research Coordinator has sufficient time to identify, consent, assess and intervene with all children receiving CHD treatment.

8.3 Efficacy Outcome Assessment and Analyses

The same graduate student, blind to study group allocation, will conduct all assessments at each visit. To minimize family impact, baseline efficacy outcomes will be assessed 1 to 2 weeks prior to the CHD treatment date, in conjunction with the child's pre-treatment clinic visit. Subsequent assessments will occur during week 7 post-treatment (post-op/post-cath visit and when activity restrictions for acute post-operative recovery end), 6 months post-baseline (post-cath visit and end of the intervention) and 12 months post-baseline (annual clinic visit; 6 months without intervention).

Motor Development: The Peabody is comprised of 6 standardized subtest scores which each have a mean score of 10 points: reflexes (child<12 months of age only), locomotor, stationary, grasping, object manipulation and visual motor integration. Gross motor (object manipulation + locomotor + stationary (+reflexes if <12 months of age); mean 30 or 40 points), fine motor (visual motor + grasping, mean 20 points) and total motor quotients (gross + fine; mean 50 or 60 points) are calculated as scores standardized by chronological age. As such, the motor quotients will remain the same over time if the child is acquiring skills at a developmentally appropriate rate. If the child's skills increase more rapidly than would be expected due to normal development the motor quotient would increase.

Demographics and Parent Perceptions: We will use the demographic questionnaire developed for our longitudinal study (HSFC grant #G-13-0002720, PI: Longmuir) to obtain parent perceptions regarding the child's physical activity participation (Appendix B). Parent reported activity restrictions will be compared to medical record activity restrictions as an index of overprotection. Scoring of the PedsQL, Social Skills Checklist, ITQOL-47 and Parenting Stress Index will follow published procedures. The exit interviews will be audio-recorded and transcribed for inductive qualitative analysis to better understand their perceptions, barriers and facilitators to study completion.

Physical Activity: At least 3 weekdays and 1 weekend day of valid data (at least 5 hours of recorded movement data not including sleep and nap times[58]) will be required[59]. Although some movement detected by the accelerometer will be attributable to the child's caregiver (e.g., lifting the child or walking while carrying the child), the impact of caregiver movement should be similar in both intervention and control groups. In our studies, accelerometer malfunction occurs in 1% of assessments and < 20% of families forget the monitor on one or more dates, although 24-h wear time minimized this risk in our previous study (HSFC grant #G-13-0002720,

PI: Longmuir). To date, all families in our studies have been willing to replace missing data by repeating the monitor measurements within 1 to 2 weeks, ensuring a complete data set for analysis.

Frequency tabulations and descriptive statistics (mean±SD or median [q1, q3] as appropriate) will describe study participants. Logistic regression will evaluate study visit when (week 7/6 mos/12 mos) age-appropriate movement skills (Peabody Total Score < 1 SD from mean) are achieved adjusting for age, sex and intervention/control. Repeated measures ANOVA will evaluate change in physical activity, adjusting for age, sex and study group (intervention/control). Change in physical activity and motor skill effect sizes by study group will indicate potential intervention impact, be used to calculate sample size for a fully-powered RCT, and confirm that changes in outcome variables are in the expected direction.

8.4 Protocol Deviations

Any and all deviations will be reported to the REB in a timely manner.

9. Data Handling and Record Keeping

9.1 Data Management Responsibilities

Personal Health Information will **not be released externally** and will be stored securely at all times. Mobile devices (e.g., laptops, USB keys, PDAs) that contain study information will be stored securely. Study data stored on these devices will be de-identified as much as possible. Electronic files stored on mobile devices will be password-protected, and encrypted.

9.2 Confidentiality & Privacy Protection

The data collected through this study will be confidential. All conditions of Bill 49, the Municipal Freedom of Information and Protection of Privacy Act 1989 and the Tri-Council Policy Statement on Research Ethics (Version 2) will be respected. Research participants will be identified only by a coded subject identification number that is not linked to personal identity. ID codes will not be based on date of birth, ethnicity, hospital record number or residency. All hard copies of the research study data will be stored in a locked cupboard in a locked office in the CHEO-RI (research data). Electronic copies of the research data will be password protected and stored on the CHEO-RI internal computer system (REDCap or HALO drive). Only the research team will have access to the research data. The list linking subject identification numbers to the research study participants (e.g., hospital unique number used to retrieve data from the medical record) will be kept separate in a password-protected and encrypted document. The only identifiable information collected during this study will be the child's name, parent name and contact information. This information will be kept separate from the study data, on a master list linking the study ID numbers with participant names. The names and contact information will be used to contact families during the study (e.g., to schedule assessment visits, monitor intervention compliance or to notify families if an intervention session has to be postponed due to weather). The only limits to the confidentiality of the research data will be in relation to legal proceedings requiring the data to be released, or in the event of imminent harm to self or others. The data may also be reviewed by members of the Research Ethics Board at the Children's Hospital of Eastern Ontario as part of auditing procedures to ensure study compliance with privacy laws and established Canadian research procedures.

9.3 Record Retention

Research study records will be retained for 7 years after study closure and final publication of study results. Electronically stored information will be verified and validated for accessibility and correctness every 3 years.

10. Budget & Finance

This project is funded by a 3- year grant-in-aid from the Heart and Stroke Foundation of Canada.

Budget Summary	Year 1	Year 2	Year 3	Total
Personnel	$70,000	$73,200	$76,500	$209,500
Benefits (28%)	$16,800	$17,360	$17,920	$52,080
Supplies	$12,935	$9,180	$3,530	$33,097
Travel	$0	$0	$2,000	$2,000
**Total**	**$99,735**	**$99,740**	**$99,950**	**$299,425**

11. Dissemination \ Publication Plan

This hypothesis-generating feasibility study will enable the design of a randomized controlled trial to evaluate physical activity intervention impact during CHD treatment recovery. Clinicians will learn of the feasibility results and planned RCT at a Grand Rounds presentation. All eight Canadian institutions that treat CHD will receive a written project summary. Families will receive assessment outcomes for their children and study results via the Healthier CHEO Kids web site (www.cheori.org). Scientific conferences (e.g., Canadian Cardiovascular Congress) and peer-reviewed journal publications will disseminate feasibility project results and the RCT study protocol to the academic community.

Reference List

(1) Health Canada. Perinatal health report: Congenital anomalies in Canada. 2002 Jan 1.

(2) Marelli AJ, Mackie AS, Ionescu-Ittu R, Rahme E, Pilote L. Congenital heart disease in the general population: changing prevalence and age distribution. *Circulation* 2007;115(2):163-72.

(3) Warnes CA, Liberthson R, Danielson GK et al. Task force 1: The changing profile of congenital heart disease in adult life. *J Am Coll Cardiol* 2001;37(5):1170-5.

(4) Voss C, Duncombe SL, Dean PH, de Souza AM, Harris KC. Physical activity and sedentary behavior in children with congenital heart disease. *Journal of the American Heart Association* 2017;6:e004665.

(5) Pemberton VL, McCrindle BW, Barkin S et al. Report of the national heart, lung, and blood institute's working group on obesity and other cardiovascular risk factors in congenital heart disease. *Circulation* 2010;121(9):1153-9.

(6) Latal B, Helfricht S, Fischer JE, Bauersfeld U, Landolt MA. Psychological adjustment and quality of life in children and adolescents following open-heart surgery for congenital heart disease: a systematic review. *BMC Pediatrics* 2009;9(6):10.

(7) Karsdorp PA, Everaerd W, Kindt M, Mulder BJM. Psychological and cognitive functioning in children and adolescents with congenital heart disease: A meta-analysis. *J Pediatr Psychol* 2007;32(5):527-41.

(8) Ginsburg KR. The importance of play in promoting healthy child development and maintaining strong parent-child bonds. *Pediatrics* 2007;119(1):182-91.

(9) Norozi K, Gravenhorst V, Hobbiebrunken E, Wessel A. Normality of cardiopulmonary capacity in children operated on to correct congenital heart defects. *Arch Pediatr Adolesc Med* 2005;159(11):1063-8.

(10) Telama R. Tracking of physical activity from childhood to adulthood: A review. *European Journal of Obesity* 2009;3:187-95.

(11) Bar-Or O, Rowland TW. Habitual activity and energy expenditure in the healthy child. *Pediatric Exercise Medicine: From Physiologic Principles to Health Care Application*. Champaign, IL: Human Kinetics; 2004. p. 64-7.

(12) Taylor RW, Murdoch L, Carter P, Gerrard DF, Williams SM, Taylor BJ. Longitudinal study of physical activity and inactivity in preschoolers: The FLAME study. *Med Sci Sports Exerc* 2009;41(1):96-102.

(13) Reilly JJ. When does it all go wrong? Longitudinal studies of changes in moderate-to-vigorous physical activity across childhood and adolescence. *Journal of Exercise Science and Fitness* 2016;14:1-6.

(14) Canadian Society for Exercise Physiology, Healthy Active Living and Obesity Research Group, University of Alberta, Public Health Agency of Canada, ParticipAction. Canadian 24-Hour Movement Guidelines for the Early Years (0-4 years): An integration of physical activity, sedentary behaviour and sleep. Canadian Society for Exercise Physiology; 2017.

(15) Longmuir PE, Turner JAP, Rowe RD, Olley PM. Postoperative exercise rehabilitation benefits children with congenital heart disease. *Clin Invest Med* 1985;8(3):232-8.

(16) Longmuir PE, Tremblay Mark S., Goode Robert C. Postoperative exercise training develops normal levels of physical activity in a group of children following cardiac surgery. *Pediatr Cardiol* 1990;11(3):126-30.

(17) Stieber NA, Gilmour S, Morra A et al. Feasibility of improving the motor development of toddlers with congenital heart defects using a home-based intervention. *Pediatr Cardiol* 2012;33(4):521-32.

(18) Klavora P. *Foundations of Exercise Science*. Toronto, Ontario: Sport Books Publisher; 2004.

(19) Centers for Disease Control. Physical activity for everyone: The importance of physical activity. 2005; Available at: URL: http://www.cdc.gov/nccdphp/dnpa/physical/importance/index.htm. Accessed August 9, 2007.

(20) Timmons BW, Naylor PJ, Pfeiffer KA. Physical activity for preschool children - how much and how? *Applied Physiology Nutrition and Metabolism* 2007;32:S122-S134.

(21) Public Health Agency of Canada. The Benefits of Physical Activity: For Children/Youth. *Public Health Agency of Canada* 2005; Available at: URL: http://www.phac-aspc.gc.ca/hp-ps/hl-mvs/pa-ap/02paap-eng.php. Accessed November 9, 2008.

(22) Malina R. Benefits of activity from a lifetime perspective. Champaign, IL: Human Kinetics; 1994.

(23) Donnelly JE, Lambourne K. Classroom-based physical activity, cognition, and academic achievement. *Prev Med* 2011 January 6;52(Suppl 1):S36-S42.

(24) Moola F, McCrindle BW, Longmuir PE. Physical activity participation in youth with surgically corrected congenital heart disease: Devising guidelines so Johnny can participate. *Paediatrics and Child Health* 2009 January 3;14(3):167-70.

(25) Borkhoff CM, Heale LD, Anderson LN et al. Objectively measured physical activity of young Canadian children using accelerometry. *Appl Physiol Nutr Metab* 2015;40(12):1302-8.

(26) Duppen N, Takken T, Hopman MTE et al. Systematic review of the effects of physical exercise training programmes in children and young adults with congenital heart disease. *Int J Cardiol* 2013;168(3):1779-87.

(27) Pate RR, Pratt M, Blair SN et al. Physical activity and public health: A recommendation from the Centers for Disease Control and Prevention and the American College of Sports Medicine. *Journal of the American Medical Association* 1995;273(5):402-7.

(28) Hovels-Gurich HH, Konrad K, Skorzenski D et al. Long-term neurodevelopmental outcome and exercise capacity after corrective surgery for tetralogy of Fallot or ventricular septal defect in infancy. *Ann Thorac Surg* 2006;81(3):958-66.

(29) Bellinger DC, Wypij D, duPlessis AJ et al. Neurodevelopmental status at eight years in children with dextro-transposition of the great arteries: The Boston Circulatory Arrest Trial. *J Thorac Cardiovasc Surg* 2003;126(5):1385-96.

(30) Bellinger DC, Wypij D, Kuban KC et al. Developmental and neurological status of children at 4 years of age after heart surgery with hypothermic circulatory arrest or low-flow cardiopulmonary bypass. *Circulation* 1999;100(5):526-32.

(31) Hovels-Gurich H, Seghaye MC, Dabritz S, al. E. Cognitive and motor development in preschool and school-aged children after neonatal arterial switch operation. *J Thorac Cardiovasc Surg* 1997;114:578-85.

(32) Hesz N, Clark EB. Cognitive development in transposition of the great vessels. *Arch Dis Child* 1988;63(2):198-200.

(33) McCrindle BW, Williams RV, Mitchell PD et al. Relationship of patient and medical characteristics to health status in children and adolescents after the Fontan procedure. *Circulation* 2006;113(8):1123-9.

(34) Fredriksen PM, Mengshoel AM, Frydenlund A, Sorbye O, Thaulow E. Follow-up in patients with congenital cardiac disease more complex than haemodynamic assessment. *Cardiol Young* 2004;14(4):373-9.

(35) Kavey RE, Allada V, Daniels SR et al. Cardiovascular risk reduction in high-risk pediatric patients: a scientific statement from the American Heart Association Expert Panel on Population and Prevention Science; the Councils on Cardiovascular Disease in the Young, Epidemiology and Prevention, Nutrition, Physical Activity and Metabolism, High Blood Pressure Research, Cardiovascular Nursing, and the Kidney in Heart Disease; and the Interdisciplinary Working Group on Quality of Care and Outcomes Research: endorsed by the American Academy of Pediatrics. *Circulation* 2006;114(24):2710-38.

(36) Lee J, Jackson J, Patel B et al. Mental health and quality of life concerns among children with cardiac diagnoses: A mixed-methods approach to identify patients at risk, sources of distress and desired support. *Health and Quality of Life Outcomes* 2019; In review.

(37) Centers for Disease Control. Physical activity and health: The benefits of physical activity. 2008; Available at: URL: http://www.cdc.gov/physicalactivity/everyone/health/index.html. Accessed November 9, 2008.

(38) Longmuir PE, Russell JL, Corey M, Faulkner G, McCrindle BW. Factors associated with the physical activity level of children who have the Fontan procedure. *Am Heart J* 2011;161(2):411-7.

(39) Longmuir PE, Banks L, McCrindle BW. Cross-sectional study of motor development among children after the Fontan procedure. *Cardiol Young* 2012;22:443-50.

(40) Massin MM, Hovels-Gurich HH, Gerard P, Seghaye MC. Physical activity patterns of children after neonatal arterial switch operation. *Ann Thorac Surg* 2006;81(2):665-70.

(41) Reybrouck T, Mertens L. Physical performance and physical activity in grown-up congenital heart disease. *Eur J Cardiovasc Prev Rehabil* 2005;12(5):498-502.

(42) Rhodes J, Curran TJ, Camil L et al. Impact of cardiac rehabilitation on the exercise function of children with serious congenital heart disease. *Pediatrics* 2005;116(6):1339-45.

(43) Longmuir PE, Tyrrell PN, Corey M, Faulkner G, Russell JL, McCrindle BW. Home-based rehabilitation enhances daily physical activity and motor skill in children who have undergone the fontan procedure. *Pediatr Cardiol* 2013;34(5):1130-51.

(44) McCrindle BW, Williams RV, Mital S et al. Physical activity levels in children and adolescents are reduced after the Fontan procedure, independent of exercise capacity, and are associated with lower perceived general health. *Arch Dis Child* 2007;92(6):509-14.

(45) Bar-Mor G, Bar-Tal Y, Krulik T, Zeevi B. Self-efficacy and physical activity in adolescents with trivial, mild, or moderate congenital heart malformations. *Cardiol Young* 2000;10(6):561-6.

(46) Rhodes J, Curran TJ, Camil L et al. Sustained effects of cardiac rehabilitation in children with serious congenital heart disease. *Pediatrics* 2006;118(3):e586-e593.

(47) Longmuir PE, McCrindle BW. Physical activity restrictions for children after the Fontan operation: Disagreement between parent, cardiologist and medical record reports. *Am Heart J* 2009;157(5):853-9.

(48) Metcalf B, Henley W, Wilkin T. Effectiveness of intervention on physical activity of children: systematic review and meta-analysis of controlled trials with objectively measured outcomes. *Br Med J* 2012;345:e5888.

(49) Duppen N, Etnel JR, Spaans L et al. Does exercise training improve cardiopulmonary fitness and daily physical activity in children and young adults with corrected tetralogy of Fallot or Fontan circulation? A randomized controlled trial. *Am Heart J* 2015;170(3):606-14.

(50) The CONSORT Group. The CONSORT Statement (Consolidated Standards of Reporting Trials). *CONSORT web site* 2010; Available at: URL: http://www.consort-statement.org/. Accessed August 22, 2019.

(51) Varni JW, Seid M, Kurtin PS. PedsQL 4.0: Reliability and Validity of the Pediatric Quality of Life Inventory Version 4.0 Generic Core Scales in Healthy and Patient Populations. *Med Care* 2001;39(8):800-12.

(52) Project DATA. Social Skills Checklist. 2007. St. Louis, University of Washington. 7-20-2019.
Ref Type: Serial (Book,Monograph)

(53) Abidin RR. *Parenting Stress Index*. 3rd ed. Odessa, Florida: Psychological Assessment Resources, Inc.; 1990.

(54) Folio MR, Fewell RR. *Peabody Development Motor Scales (PDMS-2)*. San Antonio, TX: Therapy Skill Builders; 2000.

(55) Griffiths A, Toovey T, Morgan PE, Spittle AJ. Psychometric properties of gross motor assessment tools for children: a systematic review. *BMJ Open* 2018;8:e021734.

(56) Obeid J, Nguyen T, Gabel L, Timmons BW. Physical activity in Ontario preschoolers: Prevalence and measurement issues. *Applied Physiology Nutrition and Metabolism* 2011;36(2):291-7.

(57) Adolph AL, Puyau MR, Vohra FA, Nicklas TA, Zakeri IF, Butte NF. Validation of uniaxial and triaxial accelerometers for the assessment of physical activity in preschool children. *Journal of Physical Activity and Health* 2012;9:944-53.

(58) Pfeiffer KA, Dowda M, Mciver KL, Pate RR. Factors related to objectively measured physical activity in preschool children. *Pediatr Exerc Sci* 2009;21:196-208.

(59) Trost SG, Pate RR, Freedson PS, Sallis JF, Taylor WC. Using objective physical activity measures with youth: how many days of monitoring are needed? *Med Sci Sports Exerc* 2000;32(2):426-31.

(60) Fredriksen PM, Ingier E, Thaulow E. Physical activity in children and adolescents with congenital heart disease: Aspects of measurements with an activity monitor. *Cardiol Young* 2000;10:98-106.
