# Supplementary material for: Preventing sedentary lifestyles among young children born with congenital heart defects: A feasibility study of physical activity rehabilitation after surgical or catheterization intervention
Source: PLoS One. 2023 Aug 18;18(8):e0284946. doi: 10.1371/journal.pone.0284946 (PMC10437896; doi:10.1371/journal.pone.0284946)
Supplement: S2 File — (PDF) [file pone.0284946.s003.pdf]

Activities for October/November 2022

WEEK 1 - Complete at least 3 activities each day this week. Try to choose each activity from a different focus area.

Focus of Activities: Rolling Over

**Activity**	**Instruction**
**Rattle** 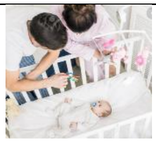	• Lay your child on their back. Shake a rattle or a noisy toy above your child's head to grab their attention. Slowly move the toy to their right side and then lower it to the ground. See if your child follows the rattle as they roll over onto their side and reach for it. If they do not do this, help your child by gently pulling their left leg over their right leg. Try on the left side!
**Rolly Polly** 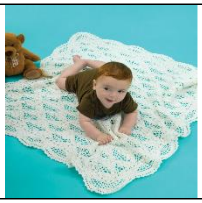	• Place your child lying on their back on a blanket. Grab both sides of the blanket and slowly rock your child back and forth. As you move the towel from side to side sing the words "Rolly polly olly!".<br>• Rock your child side to side singing the song, see if they can roll over to each side.

Focus of Activities: Sitting Up

**Activity**	**Instruction**
**Building Strength for Sitting** 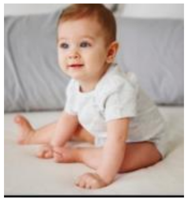	• During the next bath time, sit your child in the bath in a sitting position. Place toys around them and encourage your child to reach for the toys, placing their hands at the bottom of the bathtub to support their sitting. Hold your child around their hips during this, but offer little support so they have to use their own muscles to support their sitting, if possible.<br>• Next time you are dressing your child, support them upright in a sitting position, offering a little amount of support so they gain their own balance. Encourage them to help you dress them by putting one sock on each side of their knees on the ground and have them reach for it and give it to you. Guide their hand in doing this!
**Toy Basket** 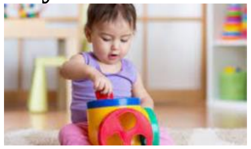	• Sit your child on the ground, as you support them sitting up, facing a basket of their favourite toys.. Allow them to feel around in the basket. See if they will grab their favourite toy from the basket. Allow them to grab onto the basket for support if they need it to sit up independently. Once they grab a toy, encourage your child to hold the toy and play with it as long as they can.<br>• Sing a song to the jingle that the noisy toy makes!

Activities for October/November 2022

Focus of Activities: Playing with Cubes

Activity	Instruction
**Play Bin** 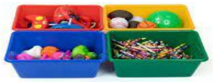	• Find a medium sized box or bin. Inside, place all kinds of toys that are small (2 to 4 inches is best). Then, sitting with your child, show them how to pick out the toys from the bin to play with by only grabbing them with the thumb and first two fingers. See how many toys they can pull out and play with this way!
**Building Blocks** 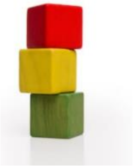	• Lay your child on their stomach with a bunch of small building blocks/cubes in front of them. If you do not have these at home, use small containers, or anything that is light weight and can easily stack on top of one another. Sit in front of your child as they are lying on their stomach, and show your child how to build a castle with the blocks. Assist them in reaching forward and grabbing the blocks and making a castle with them. Or have them knock down your castle!

Focus of Activities: Touching & Grasping Pellets

Activity	Instruction
**High Chair Games** 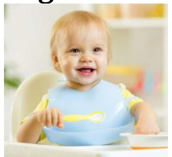	• While your child is in their high chair, place some food pellets or food puffs on their tray and encourage them to grab the pellets and eat them as a yummy snack! Show your child how you grab the pellet first with your fingers. Encourage your child to pick up at least two pellets, one at a time.<br>• Play air plane with your child's food pellets. Once they grab the pellet from their tray using their fingers, take their arm and make air plane noises as you move their arm around in the air before bringing their hand to their mouth.
**Practicing Grasping** 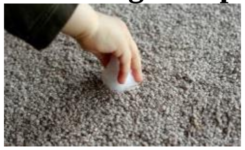	• Sit your child on your lap, facing a table. Place some cotton balls on the table in front of your child's visual field. Pretend the cotton balls are snow balls, just like the ones your child can see outside! Encourage your child to pick up the snow balls one at a time with their fingers, and throw them around like a snow ball!

Activities for October/November 2022

WEEK 2 - Complete at least 3 activities each day this week. Try to choose each activity from a different focus area.

Focus of Activities: Rolling Over

Activity	Instruction
**Twisting Baby** 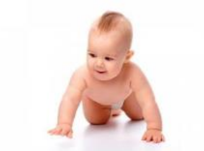	• Lay your child on their back and grab their lower trunk. Start twisting your child's body to the left then to the right as you are rocking them in a boat, while singing "Row, row, row your boat gently down the stream, merrily, merrily, merrily, merrily, life is about a dream". • Twist your child enough so that they are encouraged to roll onto their stomach from their back. Complete them while rolling from both sides.
**Monkeys on the Bed** 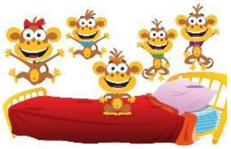	• Lay your child on their back on your bed. Sing the song "two little monkeys jumping on the bed" as you sing "one fell off and bumped his head" take your child's left arm and gently pull them onto their right side, so they roll from their back to their right side. Continue singing "Mama called the doctor and the doctor said, no more monkeys jumping on the bed!". • Repeat this on your child's other side, pulling your child's right arm over to their left side so they roll from their back onto the left side of their body.

Focus of Activities: Sitting Up

Activity	Instruction
**Huggy Stuffy** 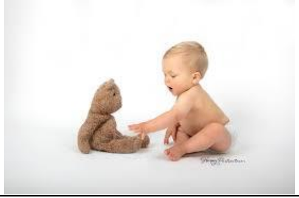	• Have your child sitting up on your lap for initial support facing away from you. You can either sit with them on the ground or against a table. Take a stuffed toy and dangle it ahead of them so that they must lean off of you and support themselves to get the toy. See if they will place their hand on the ground for support, while reaching for their toy with the other hand. • If they grab it, let them play with it until they start to lean on you again. Then repeat this activity as they reach for their toy again.
**Where'd it go?** 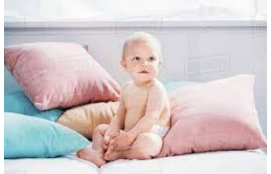	• Support your child in an upright sitting position. Have them sitting in between your legs, facing away from you. On the ground in front of them, lay out a bunch of their favorite toys. On the count of 3, cover the toys with a blanket and then say "Oh, where'd it go?" to your child. Show your child how to remove the blanket to uncover the toys and then encourage them to remove the blanket themselves!

Activities for October/November 2022

Focus of Activities: Playing with Cubes

Activity	Instruction
**Grasping Cups** 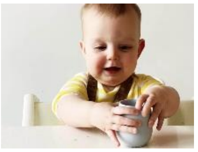	• Sit your child on your lap and have some colourful cups with handles on a table in front of you. Pick some of the cups up by their handle in front of your child and hit them together to make some noise. Guide your child in picking up the cups and help them hit the cups together to make noise. You can also make a rattle by putting bells or rice into a small plastic container. Make sure the lid is tight so that your child cannot get the small objects out.<br>• Sing a song along to the jingle your child makes!
**Cubed Food** 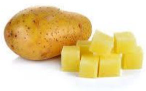	• Time for snacks again! This time, prepare some snacks in the shape of a cube such as boiled cubed potatoes or carrots, cubed avocado or even cut up pieces of bread. When picking up the pieces of food, show your child how to grab each tasty bite by only using their thumb and first two fingers.

Focus of Activities: Touching & Grasping Pellet

Activity	Instruction
**Puff Scavenger Hunt** 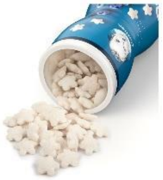	• Bring your child to a play area, where it is okay to make a little bit of a mess! Place your child in a sitting position. Grab some of your child's favorite puffs and place them all around your child on the floor, in reaching distance. Tell your child to "find the puffs" and see if they will search around and grab them! Point out the puffs to your child, and encourage them to reach for and grasp the puffs with their fingers.
**Playing with Textured Surfaces** 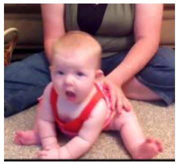	• Sit your child on the ground in front of a rug. Hold your child by their trunk and lean them forward so they put their hands on the rug and feel the rug.<br>• Put some textured paper such as wax paper, aluminum foil or construction paper in front of your child as they sit on the floor. Help them lean forward to touch the paper and feel it. See if they can crinkle it up into a snow ball!

Activities for October/November 2022

WEEK 3 - Complete at least 3 activities each day this week. Try to choose each activity from a different focus area.

Focus of Activities: Rolling Over

Activity	Instruction
**Filling up the Basket** 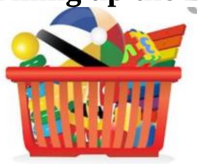	• Grab an empty basket and your child's favorite blocks or toys. Scatter the blocks or toys on the right side of your child and put the basket on the left side of your child. You can help your child to roll back and forth to get a block or a toy and put it into the empty basket. If your child is rolling from their back to their right side, gently pull their left arm over to their right side. If your child is rolling from their back to the left side, gently pull their right arm over to their left side.
**Rolling with Child** 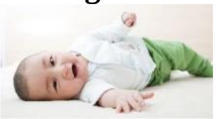	• Start with you and your child both lying on the floor on your backs beside one another. Call your child and reach toward your child and encourage your child to do the same. When your child stretches their arms toward you reward them with a hug or kisses on the hands. See if they can roll over to you from their back to their left and right sides to get a hug or a kiss!

Focus of Activities: Sitting

Activity	Instruction
**Roller Coaster** 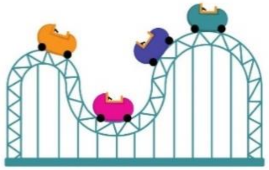	• Sit your child on your lap while sitting on a surface like a couch, facing them away from you. Support them around their lower trunk. Slowly start to bounce your child gently, telling them that the roller coaster is taking off. Every time the roller coaster is about to make a turn, tilt the child to the left and then to the right about 45 degrees. Help the child extend their arm to the tilted side while doing this. Extend their arm away from their body and land their palm face down on the surface you're on.<br>• Repeat these tilts multiple times, each time giving less assistance with extending the child's arm. Show the child that the only way to stop the roller coaster from titling is to stop it with their hand and arm.
**Patty Cake** 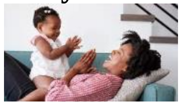	• Sit your child on a blanket in a sitting position with pillows around them for support. Sit directly in front of your child and play patty cake. Show your child that they must extend their arms out to yours to clap each other's hands. If your child is struggling with this, have another adult sit behind your child and extend their arms out for them when it is time to clap hands with you.<br>• Sing the song "Patty cake, patty cake, bakers man, bake me a cake as fast as you can, roll it, pat it, mark it with a B, put it in the oven for baby and me!" while you play this game with your child.

Activities for October/November 2022

Focus of Activities: Playing with Cubes

Activity	Instruction
**Muffin Tray** 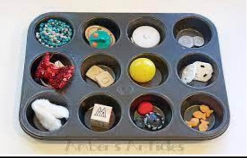	• It's time to bake some pretend muffins using little toys and a muffin tray! Start by making a small pile of items that can fit inside the holes of a muffin tray. Then, with your child, fill up the tray full of items as if you are putting together all of the ingredients to bake muffins. Once you are done, empty it out and fill it up again to make even more muffins!
**Building Structures** 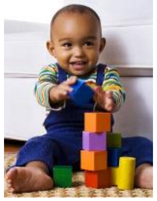	• Place some empty milk or juice cartons at your child's feet. Encourage your child to build a castle with these cartons. See if your child can bring both of their hands together in front of their chest to grab the cartons and build a structure.<br>• Place one block in your child's hand and place the second block at their feet while they are sitting. Show them you want them to pick up the other block and hit the two together to make noise.<br>• Give your child some larger building blocks and encourage them to grab the blocks with two hands and create a tall structure (assist with this) and then knock the tower down while saying "boom!"

Focus of Activities: Touching & Grasping Pellet

Activity	Instruction
**Grasping Pellets** 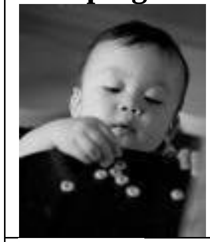	• Give your child a few pieces of cereal or some puffs as a snack. Put the cereal/puffs in a bowl without milk, and encourage your child to eat independently.<br>• Practice counting with your child by moving pieces of cereal on a plate or the high chair tray. Encourage your child to move the pieces as you count.<br>• Make shapes from pieces of cereal. You can shape the pieces to make letters or objects your child can recognize (e.g., car). Give your child pieces of cereal and ask your child to make you a picture, or make a picture together. Encourage your child to grasp the cereal with their thumb and first few fingers.
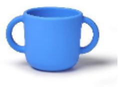 **What's in the cup?**	• Place some food pellets or puffs into a small cup. Shake around the cup in front of your child, creating a jingle! See if your child will get curious about what is in the cup, and let them reach in and grasp the pellets with their thumb and index finger!

Activities for October/November 2022

WEEK 4 - Complete at least 3 activities each day this week. Try to choose each activity from a different focus area.

Focus of Activities: Rolling Over

Activity	Instruction
**This Little Piggy** 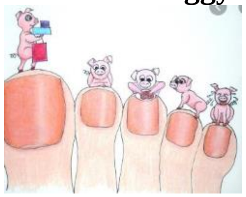	• Start with your child lying on their back. Hold your child's left hand and gently stretch their arm out until it is straight. Then sing the nursery rhyme "This little piggy went to market" on the child's fingers while holding the arm out straight, gently pulling their left hand over so they roll onto the right side of their body as you sing "wee wee wee all the way home".<br>• The words for the song are "This little piggy went to the market, and this little piggy went home, this little piggy had roast beef and this little piggy had none and this little piggy went wee wee wee all the way home.<br>• Repeat this activity on your child's left side!<br>• Repeat this activity with your child's legs so that you pull their leg over to stabilize them on their side.
**Tug of War** 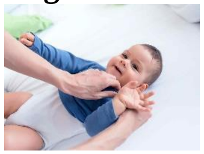	• Have a playful tug-of-war with your child, while you are both laying on your backs. Give your child a scarf to hold onto. Gently pull on the scarf and then encourage your child to pull back the other way. Eventually, pull the scarf hard enough that your child rolls from their back to one side of their body!<br>• Repeat this on the other side of their body. Be careful you do not pull the scarf too hard, and provide assistance to your child if they need it

Focus of Activities: Sitting

Activity	Instruction
**Making Music** 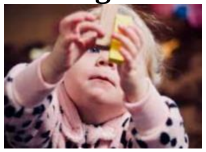	• Place your child in a sitting position. Give your child two items to hold and encourage them to bang these two items together to create some noise. You can give them two blocks, two cups or two small containers. Guide their hands in doing this at first, then see if they can sit independently and make some noise!

Activities for October/November 2022

**Grabbing Toys** 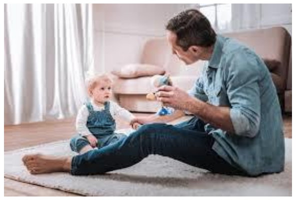	• Place your child in a sitting position on a blanket with their favourite toy on the floor in front of them. Once they focus on the toy, release your support on them and encourage them to grab the toy by saying "get the toy" and hold onto it while sitting up. Complete this activity until they hold the toy while maintaining balance while sitting for thirty seconds. However, provide assistance if they need it. Move the toy around in different spots so they are reaching in different locations.

Focus of Activities: Playing with Cubes

**Activity**	**Instruction**
**Pick Up Two** 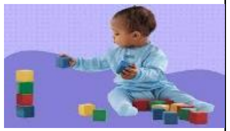	• Once your child is comfortable picking up one block or a small toy, encourage your child to pick up two at a time using the same hand.<br>• Start with a large container or one that will make a sound when the object hits the bottom. Let the child enjoy dumping the cube/toys out of the container then encourage your child to pick them up again.
**Five Little Ducks** 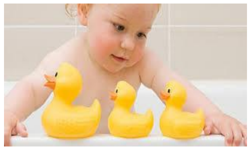	• Float five small toys in the bathtub with your child. Pretend that each toy is a baby duck. Have a bigger toy or a rubber duck to be the Mommy duck. Sing the "five little ducks" song and encourage your child to pick up one of the little ducks for each verse. The words of the song are "Five little ducks went swimming one day, over the pond and far away. Mamma duck said quack quack quack only four little ducks came back. Four little ducks went swimming one day, over the pond and far away. Mama duck said quack quack quack and only three little ducks came back … etc.

Focus of Activities: Touching & Grasping Pellets

**Activity**	**Instruction**
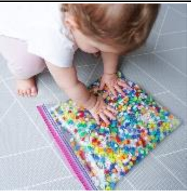 **Mess Free Water Play**	• Create a sensory bag for your child. Fill a zip-lock bag up with water and place small beads in the bag. Place your child in their highchair and place the bag on the tray in front of them. Encourage your child to practice grasping at the bag, trying to grasp the beads with their thumb and index fingers!
